# A dataset of consumer perceptions of gustometer-controlled stimuli measured with three temporal sensory evaluation methods

**DOI:** 10.1016/j.dib.2023.109271

**Published:** 2023-05-30

**Authors:** Noëlle Béno, Léna Nicolle, Michel Visalli

**Affiliations:** a Centre des Sciences du Goût et de l'Alimentation, AgroSup Dijon, CNRS, INRAE, Université Bourgogne, F-21000 Dijon, France; b INRAE, PROBE research infrastructure, ChemoSens facility, F-21000 Dijon, France

**Keywords:** Free-comment, Temporal dominance of sensations, Temporal check-all-that-apply, Attack-evolution-finish, Rate-All-That-Apply, Sensory perception, Taste interaction, Sensometrics

## Abstract

This paper describes data on the consumer sensory perception of liquid mixtures including sapid and aromatic compounds. A total of 149 consumers participated in this study. They were randomly assigned to one of three panels. Each panel used a different temporal sensory evaluation method among Temporal Dominance of Sensation (TDS, *n* = 50), Temporal Check-All-That-Apply (TCATA, *n* = 50) and Attack-Evolution-Finish Rate-All-That-Apply (AEF-RATA, *n* = 49) to evaluate solutions delivered by a gustometer (Burghart GU002). First, four simple solutions (composed of a single compound) were delivered to the consumers to evaluate their recognition ability using Free Comment. Second, eighteen complex solutions (composed of two to five compounds varying in their sequence, intensity and duration of stimulation) were delivered to the consumers to evaluate their ability to use the three temporal evaluation methods. The compounds included sodium chloride (“salty”), saccharose (“sweet”), citric acid (“acid”), citral (“lemon”) and basil hydrosol (“basil”). The data were used to assess the validity and reliability of the temporal sensory methods in an article entitled “Assessment of the validity and reliability of temporal sensory evaluation methods used with consumers on controlled stimuli delivered by a gustometer". The data could be reused by researchers interested in studying the effect of interactions between sapid and aromatic compounds on perception.

**Specifications Table**

**Table:** 

Subject	Food science
Specific subject area	Temporal sensory evaluation
Type of data	Table Figure
How the data were acquired	Tree consumer panels evaluated liquid solutions using each a different temporal sensory evaluation method among Temporal Dominance of Sensation (TDS [1], *n* = 50), Temporal Check-All-That-Apply (TCATA [2], *n* = 50) and Attack-Evolution-Finish Rate-All-That-Apply (AEF-RATA, *n* = 49), an adaptation of AEF-Applicability [3]. Experiences were carried out individually in the human olfaction/taste laboratory of the ChemoSens platform. The solutions were delivered by a gustometer Burghart GU002 at a constant flow rate of 350 µL/s. The data were recorded using the TimeSens© [4] software, version 2.0.
Data format	Tables in raw format (XLSX file) Questionnaire (PDF)
Description of data collection	**Recognition task** Four single isointense compound solutions were delivered to the consumers: two sapid compounds over three (sodium chloride, saccharose, citric acid), one aromatic compound over two (citral, basil hydrosol), plus one replicate. The solutions were presented according to a William's latin square design balanced at the panel level. The solutions were delivered during eight seconds. The durations of perception were recorded, and the consumers had to self-report the sensation(s) they perceived using Free-Comment. **Temporal perception task** Eighteen (14 different and four replicated) multi-compound solutions were delivered to the consumers in the same order. They were composed of two to five compounds (same compounds as for the recognition task), varying in their sequence (with or without overlap), intensity (three isointense concentration levels: weak, medium, and strong) and duration of stimulation. Each sequence lasted 30 s. The same list of eight attributes was used in TDS, TCATA and AEF-RATA. This list includes five attributes corresponding to the compounds: sweet, salty, acid, lemon and basil; and three distractors: bitter, licorice, and mint. For TDS and TCATA, the record of the perception started when the consumers clicked on an attribute. The times and durations of perception of dominance (TDS) and applicability (TCATA) were thus recorded. For AEF-RATA, the consumers had to retrospectively rate the intensity they perceived (weak, medium, strong) for each applicable attribute and for each of three periods: “at the beginning”, “after a few seconds”, “at the end”.
Data source location	Institution: INRAE City/Town/Region: Dijon Country: France
Data accessibility	Repository name: Mendeley data Data identification number: 10.17632/3j6h7mrxnf.1 Direct URL to data: https://data.mendeley.com/datasets/3j6h7mrxnf/1
Related research article	

## Value of the Data

• These data are useful because they allow a better understanding of what is actually measured with temporal evaluation methods by making it possible to compare the declarative results relating the sensory perception of consumers with the chemical reality of the controlled stimuli delivered using a gustometer.
• The food science and sensometric community can benefit from these data to assess the construct and criterion validity of concepts related to sensory evaluation methods (e.g. dominance vs. applicability) and their derived measurements (e.g. intensities vs. durations vs. citation rates, periods vs. continuous time, etc.)
• These data can also be reused to compare and document the performances of temporal sensory evaluation methods (e.g. repeatability, discrimination) or to develop new statistical analyses better suited to the specific nature of temporal measurements.
• These data can also be reused by researchers interested in studying the effects of congruent and incongruent interactions between sapid and aromatic compounds on the sensory perception of consumers (e.g. when acid and lemon compounds are delivered at the same moment).

## 1 Objective

These last years, numerous sensory evaluation methods have been developed to capture the dynamic of perception during the tasting of food products. However, “sensory reality” remains unknown, thus it is difficult for sensory scientists to determine which method is more valid. The data were collected to investigate how the perception of controlled temporal stimuli delivered using a gustometer are transcribed by consumers using three different sensory evaluation methods: Temporal Dominance of Sensations (TDS), Temporal Check-All-That-Apply (TCATA), and Attack-Evolution-Finish Rate-All-That-Apply (AEF-RATA). The ultimate objective was to confront the results of the methods to the chemical reality of the stimuli to document the validity and reliability of the methods.

## 2 Data Description

The dataset is provided as an Excel file (data.xlsx) including six sheets.

***Consumer*** provides information about the participants.

“Panel”: name of the panel to which the participant has been assigned. The name of the panel corresponds to the name of the method used for the temporal sensory evaluation: TDS, TCATA, and AEF-RATA.

“Consumer”: unique code of the participant.

“Gender”: gender of the participant (Male or Female).

“Age”: age of the participant.

***Stimuli*** provides information about the sequences delivered in the recognition task and in the temporal perception task.

“Stimulus”: code of the stimulus. Stimuli with the following codes have been delivered during the recognition task: Acid, Sweet, Salty, Lemon and Basil. Other codes correspond to stimuli delivered during the temporal perception task.

“Attribute” corresponds to the attribute related to the delivered compound (salty for sodium chloride; sweet for saccharose; acid for citric acid; lemon for citral, and basil for basil hydrosol).

“Time”: beginning time of delivery of “Attribute” expressed in seconds.

“Quantity”: quantity delivered at the different concentrations of the intensity levels (10% for weak; 50% for medium, and 90% for strong).

***RecognitionTask*** provides data related to the recognition task.

“Panel”, “Consumer”, “Stimulus”: see above.

“FrenchDescription”: free comments (in French) reported by the participants to describe the stimulus.

“Attributes”: sensory attributes extracted from free comments and translated to English by the experimenters.

“Result”: categorization of the result of the recognition task performed by the experimenters in five levels: not identified (NotIdentified), approximately identified with the need for several attributes (Identified_Approximate_NotUnique), approximately identified with the need for a single attribute (Identified_Approximate_Unique), identified with the need for several attributes (Identified_Exact_NotUnique) and identified with the need for a single attribute (Identified_Exact_Unique).

“Duration”: duration of perception of the stimulus, in seconds.

***TDS, TCATA*** and ***AEF-RATA*** provide data collected with the corresponding temporal evaluation methods during the temporal perception task.

“Panel”, “Consumer”, “Stimulus”: see above.

“Time”: time (in seconds) of click on the attribute (TDS and TCATA)

“Period”: period (A for Attack, E for Evolution, F for finish) during which the attribute was retrospectively declared applicable (AEF-RATA).

“Attribute”: attribute clicked by the participant (acid, basil, bitter, lemon, licorice, mint, salty or sweet) + end of perception (“stop”, TDS and TCATA).

“Score”: for TDS: dominant attributes (always 1), for TCATA: applicable attributes (1: start of applicability, 0: end of applicability), for AEF-RATA: perceived intensity for applicable attributes (1: weak, 2: medium, 3: strong).

**Fig. 1:**
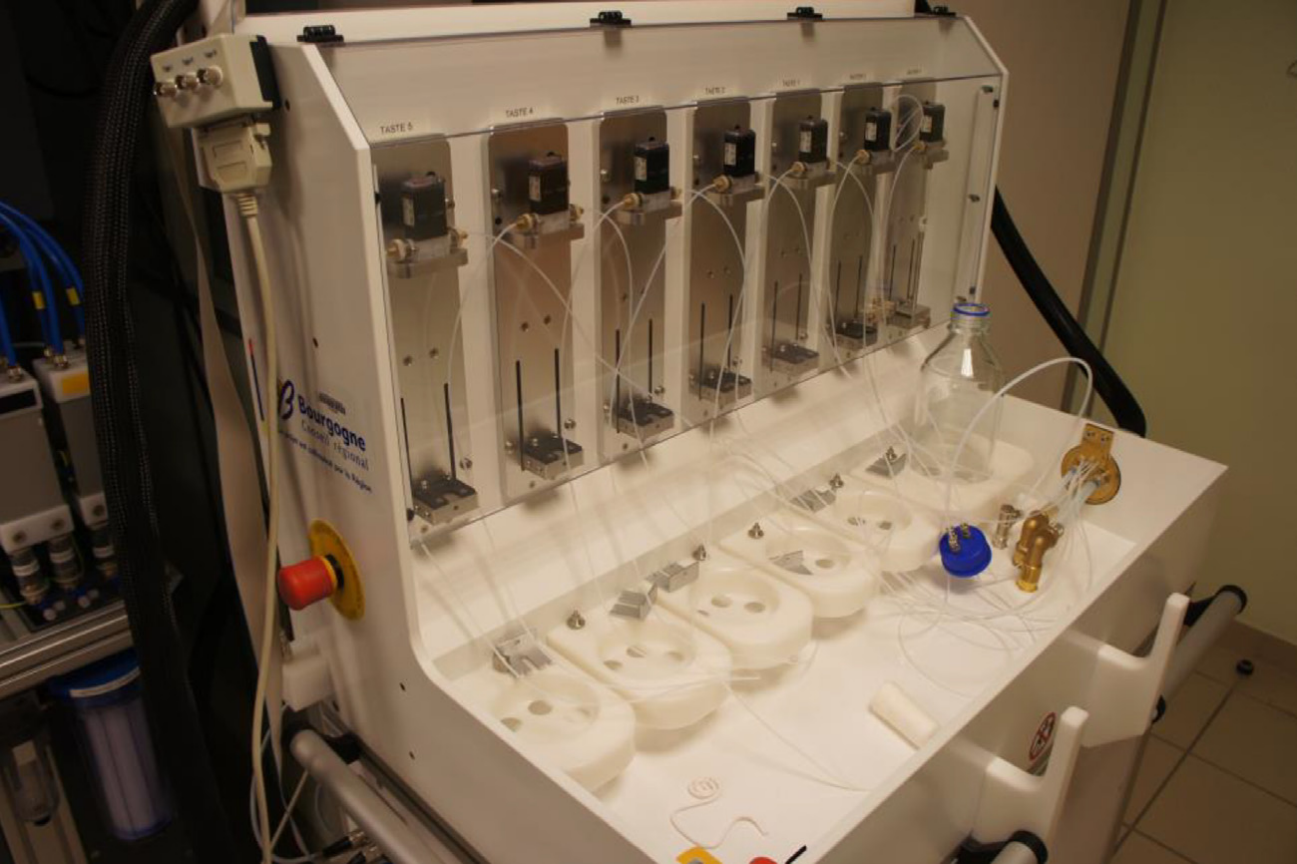
gustometer model Burghart GU002.

Fig. 1 shows a picture of the gustometer.

**Fig. 2:**
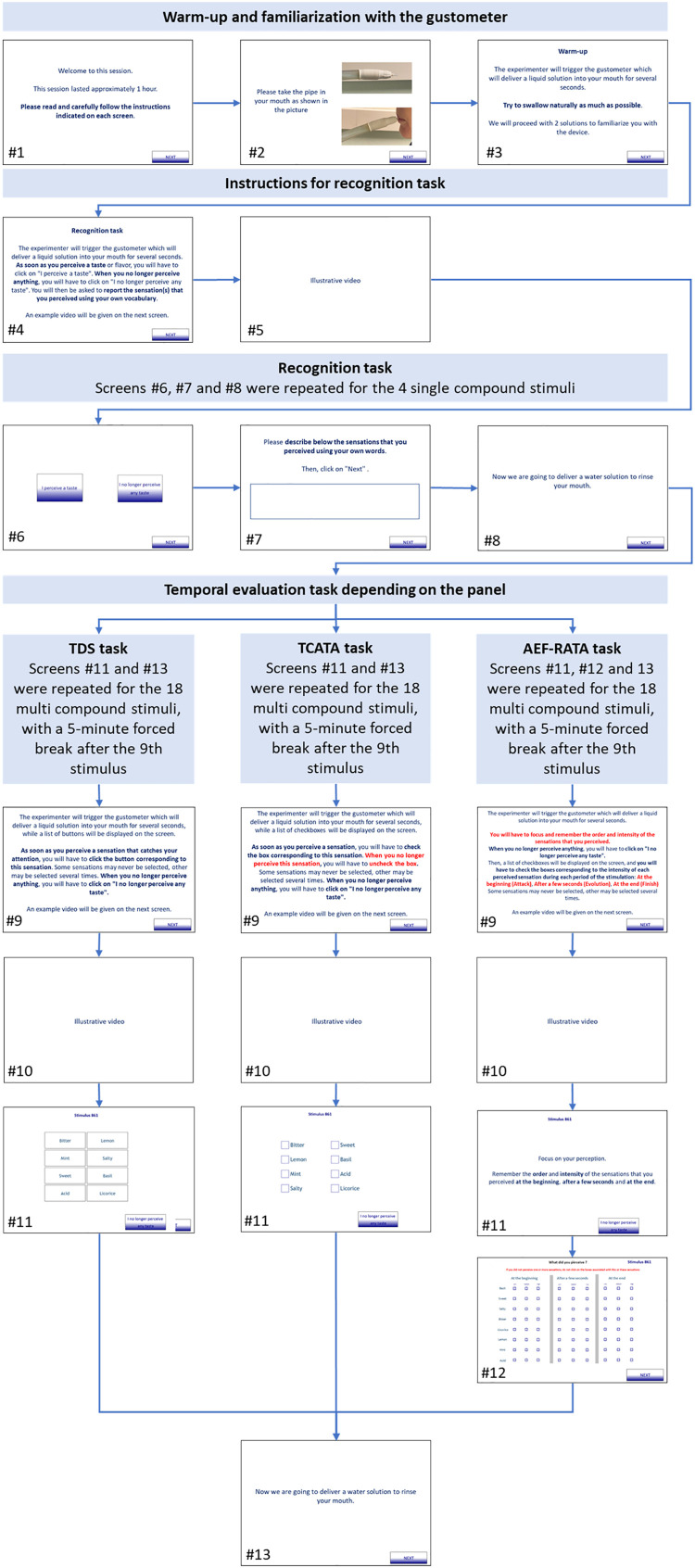
screens displayed to the consumers.

Fig. 2 shows the screens displayed to the consumers.

Table 1 describes the composition of the three consumer panels.

**Table 1:** Composition of the consumer panels.

Panel	TDS	TCATA	AEF-RATA
Average age	46.8	46.2	44.4
Gender: male	21	16	19
Gender: female	29	34	30

Table 2 describes the sapid and aromatic compounds used in the stimuli.

**Table 2:** Information about the sapid and aromatic compounds used in the stimuli, and corresponding sensory attributes.

Compound	Attribute	Raw formula	CAS Number	Lot number	Expiration date	Concentration
Sodium chloride	Salty	NaCl	7647–14–5	19,100,184/D	10–2024	25 g/l
Saccharose	Sweet	C12H22O11	57–50–1	71,616,173	–	250 g/l
Citric acid	Acidic	C6H8O7 x H20	5949–29–1	20,070,058/A	06–2023	10 g/l
Citral	Lemon	C10H16O	5392–40–5	MKCJ7159	03–2024	10 mg/l
Basil hydrosol (Plantago)	Basil	–	–	HY2120 HY2120	08–2022 08–2023	20 g/l

## 3 Experimental Design, Materials and Methods

### 3.1 Participants

A total of 149 consumers (56 men and 93 women, between 21 and 65 years old) participated in this study. They were preselected from a population registered in the ChemoSens Platform's PanelSens database (declared to the relevant authority, Commission Nationale Informatique et Libertés – CNIL, authorization number 1,148,039). The inclusion conditions for participating in this study were as follows: being between 18 and 65 years old; not suffering from food or non-food allergies; not being pregnant or breastfeeding and not following a restrictive diet incompatible with the consumption of sugar or salt.

The purpose of the study was explained via an information sheet sent by email. The consumers had to fill out a written informed consent form. They were compensated for their participation (one session) with vouchers worth 10 euros.

The selected consumers were randomly assigned to one of three panels, with a constraint of balance in gender and age between panels. Each panel used a different temporal method among AEF-RATA (*n* = 49), TCATA (*n* = 50) and TDS (*n* = 50).

### 3.2 Stimuli

Different stimuli were delivered to the consumers using a gustometer model Burghart GU002.

This device consists of five syringes (2.5 ml) containing the sapid and aromatic compound solutions, and two syringes (2.5 ml) containing water. The stimuli temperature is regulated at 35 °C to avoid thermal irritations. Up to five compounds can be loaded, mixed, and diluted by the gustometer.

The selected compounds are described in Table 2. Their concentrations were chosen to be as close to isointensity as possible, based on internal pre-tests.

Controlled by a computer, the gustometer enables to deliver liquids in the form of pulses (70 µl every 100 ms) through a tube (350 µl/s flow rate). Up to 39 classes could be defined for the pulses, one class defining the selected syringe(s), the dilution of the compound, and sequences defining the duration of the class(es) delivery. The total dilution of the compounds could not exceed 100% within a class. Three classes of concentration were chosen for the compounds: weak, medium, and strong, corresponding respectively to 10, 50 and 90% of the concentrated solution.

As swallowing was made more difficult by the continuous flow of liquid, the stimulation durations should not exceed 30 s to limit the volume in the mouth to 10 ml and reduce the discomfort of consumers.

### 3.3 Data collection

The consumers participated in individual sessions of approximately one hour in the olfactometry lab of ChemoSens. They were installed in a booth and had to read and accept the condition of the study. The consumers' responses were collected using TimeSens© V2 software.

## 4 Familiarization with the Gustometer (screens #1–3)

The consumers were instructed about how the gustometer works and how to position their mouth (screen #2). They could ask all the questions they wanted to the experimenter who piloted the gustometer. Then, they were invited to experiment the stimulation with the gustometer (screen #3) so that they get used to the device, and in particular to swallow at the same time as they received a liquid flow under pressure (they were free to swallow whenever they wished). Two water solutions were delivered during 20 and 30 s.

## 5 Recognition Task (screens #4–5)

Four medium-concentration single-compound solutions were delivered to the consumers, each for 16 s at a constant flow rate of 350 µl/s: water for 4 s, compound for 8 s, then water for 4 s. The order of presentation of the stimuli follows an incomplete William's latin square design balanced at the panel level. Each consumer evaluated two sapid compounds over three (sodium chloride, saccharose, citric acid) and one aromatic compound over two (citral, basil hydrosol). The first or second solutions delivered was replicated last.

The instructions for the task were presented on the screen (screen #4), then a tutorial video was shown (screen #5). The durations of perception were recorded: the consumers had to click on a button when they started to perceive and when they no longer perceived anything (screen #6). Then, the consumers had to self-report the sensation(s) they perceived using Free-Comment (screen #7). Between two stimulations, water was delivered by the gustometer during 10 s to clean the tubes and rinse the mouths of consumers (screen #8).

## 6 Temporal Evaluation Task (screens #9–12)

Eighteen multiple-compound solutions were delivered to the consumers (14 different and 4 replicated). The order of presentation was the same for all the consumers: S01, S07, S011, S04, S12, S14, S10, S02, S013, S03, S08, S09, S06, S05, S11_2, S02_2, S08_2, S14_2. The stimuli varied in number, sequence (with or without overlap), duration, and concentration of the compounds. Each sequence lasted 30 s and 10.5 ml were delivered at a constant flow rate of 350 µl/s.

The instructions for the task were presented on the screen (screen #9), then a tutorial video was shown (screen #10). Each panel used a different temporal sensory evaluation method. The same list of eight attributes was used in TDS, TCATA and AEF-RATA. This list includes five attributes related to the compounds: sweet, salty, acid, lemon and basil; and three distractors: bitter, licorice and mint. The order of presentation of the attributes was randomized between the consumers but remained the same for a consumer within the session. For TDS and TCATA (screen #11), the chronometer started when the consumers clicked on an attribute. The times and durations of perception of dominance (TDS) and applicability (TCATA) were recorded. For AEF-RATA (screen #12), the consumers had to retrospectively rate the intensity they perceived (weak, medium, strong) for each applicable attribute and for each of three periods: “at the beginning”, “after a few seconds”, “at the end”. Between two stimulations, water was delivered by the gustometer for 10 s to clean the tubes and the rinse mouths of consumers. A five-mean break was imposed after the 9th stimulus.

## Ethics Statements

Each participant was informed of the conditions for participating and validated a consent form. The research was carried out in conformity with the Declaration of Helsinki.

## CRediT authorship contribution statement

**Noëlle Béno:** Conceptualization, Data curation, Investigation, Methodology, Project administration, Resources, Validation, Writing – original draft. **Léna Nicolle:** Data curation, Investigation, Methodology, Resources, Writing – review & editing. **Michel Visalli:** Conceptualization, Funding acquisition, Methodology, Software, Supervision, Visualization, Writing – review & editing.

## Declaration of Competing Interest

The authors declare that they have no known competing financial interests or personal relationships that could have appeared to influence the work reported in this paper.

## Data Availability

A dataset of consumer perceptions of gustometer-controlled stimuli measured with three temporal sensory evaluation methods (Original data) (Mendeley Data).
